
JOURNAL INFORMATION
==============================
NLM Title Abbreviation: Perspect Med Educ
Journal ID: Perspect Med Educ
NLM Journal ID: 101590643

Perspectives on Medical Education

ISSN: 2212-2761
EISSN: 2212-277X
Publisher: Ubiquity Press

ARTICLE INFORMATION
==============================
PMCID: PMC6382619
DOI: 10.1007/s40037-019-0494-5
Article ID: 494
Article version: 1
Subjects: Thanks to Reviewers

Thanks to reviewers

Electronic publication date: 2019 Jan 22
Print publication date: 2019 Feb
Volume: 8
Issue: 1
First page: 60
Last page: 61
Copyright: © The Author(s) 2019
License: Open Access This article is distributed under the terms of the Creative Commons Attribution 4.0 International License (http://creativecommons.org/licenses/by/4.0/), which permits unrestricted use, distribution, and reproduction in any medium, provided you give appropriate credit to the original author(s) and the source, provide a link to the Creative Commons license, and indicate if changes were made.
License URL: https://creativecommons.org/licenses/by/4.0/

issue-copyright-statement: © The Author(s) 2019

==============================
Perspect Med Educ (2019) 8:60–61
https://doi.org/10.1007/s40037-019-0494-5

THANKS TO REVIEWERS

### Thanks to reviewers

© The Author(s) 2019

The Editorial Team would like to thank all the reviewers for their time and advice which helped us to assess and improve the papers that were submitted to us in 2017. The reviewers' critical and constructive feedback greatly contributed to raising the quality of the journal.

Madeleine Abrandt Dahlgren
Aileen Ackland
Marieke Adema
Azam Afzal
Betina Andersen
Douglas Archibald
Dorene Balmer
Deirdre Bennett
P. M. Bloemendaal
Peter Boendermaker
Tobias Boerboom
Klara Bolander Laksov
Jan Borleffs
Johan Bredée
Jamiu Busari
William Bynum
Diana Callender
Holly Caretta-Weyer
Ming-Ka Chan
Teresa Chan
Jeffrey Cheung
Maitreya Coffey
Carlos Collares
Patrick Cras
Sayra Cristancho
John Cyrus
Matthew D'Angelo
Mary E. W. Dankbaar
Heather Davidson
Peter de Jong
Joy de Vries-Erich
A. Deketelaere
Friedo Dekker
Josette Denekens
Goran Devedzic
Luke Devine
Shafik Dharamsi
Marja Dijksterhuis
Diana H. J. M. Dolmans
Jeroen Donkers
Valerie Dory
Jos Draaisma
Kristina Dzara
Andem Ekpenyong
Rachel Ellaway
Rik Engbers
Christian Ezeala
Götz Fabry
Susan Farrell
Dario Fernandes
Simon Fleming
Adrian Freeman
Judith French
Paul Fullerton
Jane Gagliardi
R. O. B. Gans
Eric Gantwerker
Loretta Garvey
Simon Gay
Andrea Gingerich
Shiphra Ginsburg
Mark Goldszmidt
Holly Gooding
Gerard Gormley
Daniel Grace
F. J. M. Grosfeld
Stephen Harte
Nadia Hartman
Joshua Hartzell
Margaret Hay
Esther Helmich
Helen Henderson
Paul Hibbert
Brian Hodges
Graeme Horton
Larry Hurtubise
Cassio Ibiapina
Johanna Jacobs
Renate Kahlke
Grainne Kearney
Jan Kiesewetter
Andrew King
Benjamin Kinnear
Anne Kinsella
Karen Könings
Abigail Konopasky
W. D. J. Kremer
Frank Krings
Kulamakan Kulasegaram
Victor Lee
Janet Lefroy
Jimmie Leppink
Marcel Levi

Lorelei Lingard
Andrew Linn
Ashley Lubberdink
Mario Maas
Diana Madden
Annalisa Manca
Maria Athina Martimianakis
Shannon Martin
Anne McKee
Robert McKinley
Anne Messman
Holly Meyer
Tracy Moniz
Sandra Monteiro
Sandra Nicholson
Laura Nimmon
Bridget O'Brien
Arko Oderwald
Lotte O'Neill
Patricia O'Sullivan
Glenn Paetow
Elise Paradis
Lyndal Parker-Newlyn
Harm Peters
Nicole Piemonte
Richard Pittini
Eve Purdy
Martin Pusic
Antonia Quinn
Anne Marie Rieffestahl
Herma Roebertsen
Durga Roy
Anna Ryan
Jason Sapp
Joan Sargeant
Stefan Schauber
Susie Schofield
Stefanie Sebok
Jessica Servey
Justin Sewell
Neeral Shah
Annemarie Spruijt
Christine Stehman
Christina St-Onge
Yoyo Suhoyo
Nancy Tannery
Walter Tavares
E. W. M. T. ter Braak
Aliki Thomas
Jaap Tullekem
Michael Tutwiler
Sebastian Uijtdehaage
Sjoukje Van den Broek
Nadine van der Lee
Renee van der Leeuw
Cees van der Vleuten
Asha Venkatesh
Megan Von Isenburg
Bruce Wainman
Bjorn Watsjold
Fiona Webster
Laura Weingartner
Indah Widyahening
Tim Wilkinson
Yolande Witman
Nicole Woods
Anouk Wouters
Amy Zeidan

**Open Access** This article is distributed under the terms of the Creative Commons Attribution 4.0 International License (http://creativecommons.org/licenses/by/4.0/), which permits unrestricted use, distribution, and reproduction in any medium, provided you give appropriate credit to the original author(s) and the source, provide a link to the Creative Commons license, and indicate if changes were made.
